# A Case of Late-Onset Neutropenia in Relapsing-Remitting Multiple Sclerosis Following Ocrelizumab Therapy

**DOI:** 10.7759/cureus.51729

**Published:** 2024-01-05

**Authors:** Reema A Alabdulqader, Wafa Alnasser, Hussain J Aljubran, Hassan H Alkhulaif

**Affiliations:** 1 Internal Medicine, Imam Abdulrahman Bin Faisal Hospital, Dammam, SAU; 2 Infection Control, Imam Abdulrahman Bin Faisal Hospital, Dammam, SAU; 3 Medicine, Imam Abdulrahman Bin Faisal University, Dammam, SAU

**Keywords:** urinary tract infection, nitrofurantoin, multiple sclerosis, ocrelizumab, neutropenia

## Abstract

Ocrelizumab, a monoclonal antibody, has proven effective in treating both primary progressive and relapsing-remitting multiple sclerosis. Common adverse effects observed in clinical studies include flushing, sore throat, pruritus, and rash. This abstract discusses a case of severe, late-onset neutropenia in a patient with relapsing-remitting multiple sclerosis undergoing ocrelizumab treatment. The neutropenia emerged 46 days following the patient's most recent ocrelizumab dose. The patient responded well to treatment with intravenous meropenem and filgrastim. This rare and unforeseen adverse effect highlights the importance of regular blood monitoring for early detection of severe neutropenia in patients treated with ocrelizumab.

## Introduction

Ocrelizumab, a monoclonal antibody, effectively treats relapsing-remitting and primary progressive multiple sclerosis [1]. This drug targets CD20, a membrane glycosylated phosphoprotein predominantly found on B-lymphocytes but absent in plasma cells and neutrophils [2]. The most common adverse effects observed in clinical trials were infusion-related symptoms, including flushing, throat irritation, pruritus, and rash [3]. Additionally, rare side effects such as malignancies and infections have been reported, though their direct connection to ocrelizumab remains unconfirmed [4]. This report presents a case of late-onset profound neutropenia (LON) in a patient with relapsing-remitting multiple sclerosis treated with ocrelizumab. The patient exhibited symptoms 46 days post-infusion, including headache, subjective fever, and chills. We aim to highlight this unusual side effect, emphasize the importance of routine hematological monitoring, and inform patients about the risk of severe neutropenia.

## Case presentation

A 24-year-old female patient diagnosed with relapsing-remitting multiple sclerosis seven years prior has been receiving 600 mg of ocrelizumab intravenously every six months for the past 18 months. She reported no serious side effects from previous doses.

The patient presented to the emergency department (ED) with a severe bilateral frontoparietal headache, which began three days before her visit, accompanied by fever and chills. A week earlier, she visited the ED with symptoms indicative of a urinary tract infection. A culture identified extended-spectrum beta-lactamase (ESBL) E. coli, and she was treated with nitrofurantoin. Her vital signs were blood pressure 115/75 mmHg, heart rate 110 beats per minute, respiratory rate 20 breaths per minute, and temperature 37.7 °C. She appeared comfortable, conscious, alert, and oriented. A cardiovascular examination revealed normal heart sounds with no additional sounds. Respiratory examination showed normal vesicular breath sounds. The abdominal exam was soft and lax without tenderness, and the central nervous system exam was intact.

**Figure 1:**
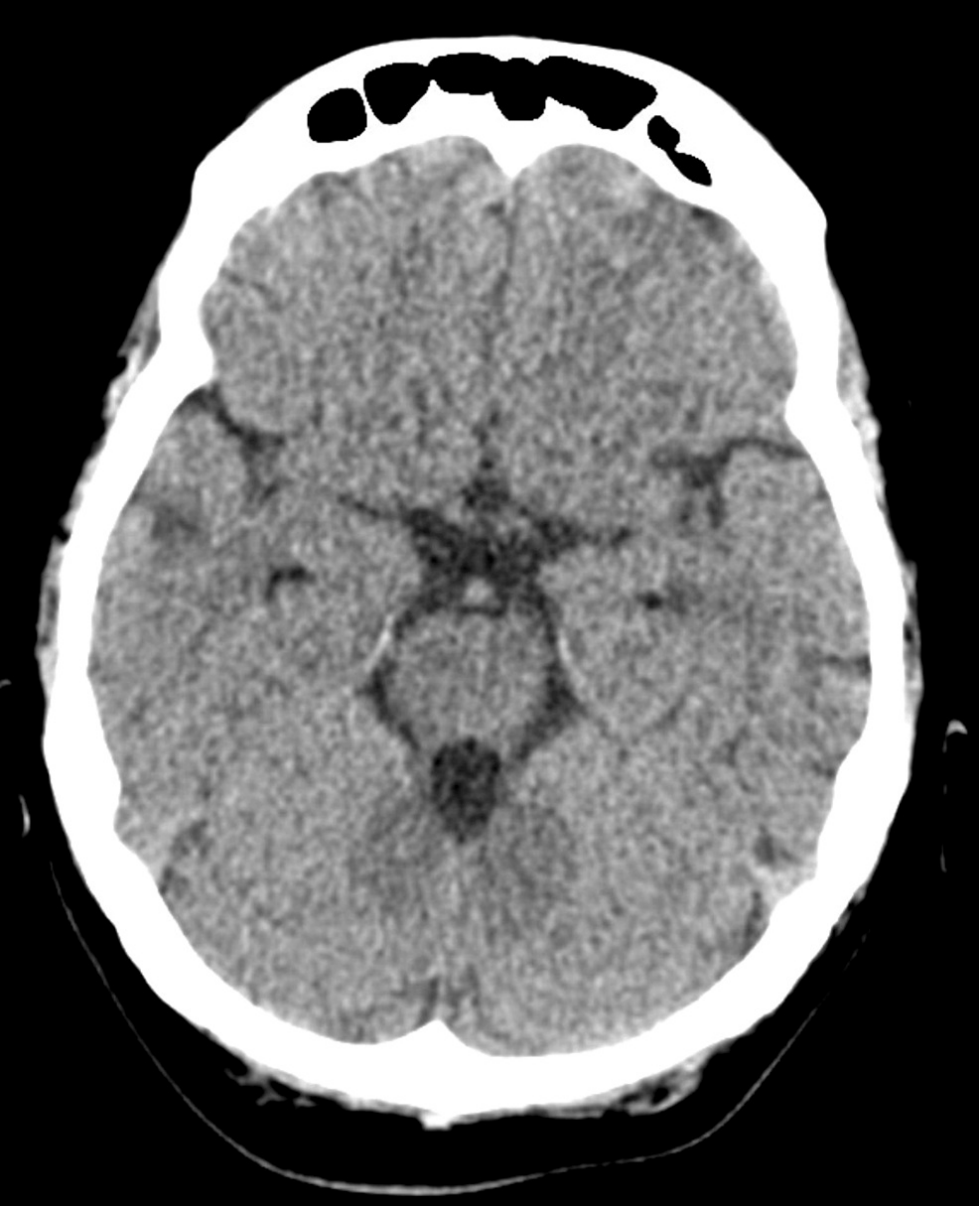
Vertical brain CT scan which revealed normal findings.

Laboratory tests showed complete blood counts: a white blood cell count of 1.1 × 10^9/L with an absolute neutrophil count (ANC) of 0.0 × 10^9/L (Table 1), normal hemoglobin and platelet counts, and unremarkable renal and liver function tests. Her brain computed tomography scan displayed changes related to multiple sclerosis but no intracerebral hemorrhage (Figure 1). Consequently, she was hospitalized for febrile neutropenia and commenced on meropenem and vancomycin. Due to hypotension being unresponsive to fluids, she was transferred to the intensive care unit later that night. Blood cultures identified gram-negative bacilli 10 hours post-admission, later confirmed as ESBL E. coli. Treatment included seven-day filgrastim (granulocyte-colony stimulating factor) and intravenous meropenem. By the third day, her neutrophil level became 2.69 x 10^9 and then normalized the days after. Serology testing to rule out other autoimmune diseases was not done. Clinically, she also showed improvement. If her ANC decreased post-discharge, a bone marrow examination was planned. However, follow-up complete blood counts two and four weeks after discharge confirmed the full recovery of her neutrophil count, and it was decided to continue on ocrelizumab.

**Table 1:** Serial blood workup of the patient from admission to discharge. WBC: White blood cell; ANC: absolute neutrophil count

Date	Analysis			
	WBC (x10^9/L)	ANC (x10^9/L)	Platelets (x10^9/L)	Hemoglobin (g/L)
20/7/2023	2	0	347	100
21/7/2023	1.7	0.03	236	85
22/7/2023	4.9	2.69	251	84
23/7/2023	18.9	15.20	297	75
24/7/2023	11.7	7.61	281	76
25/7/2023	10.2	7.35	323	75
26/7/2023	10.2	7.42	340	82
27/7/2023	10.9	8.59	376	95
28/7/2023	6	3.90	407	92
29/7/2023	5.8	3.64	453	94
Reference Range	4-11	2-7.5	150-400	160-120

## Discussion

LON is characterized as an ANC less than 1.5 × 10^9/L, first appearing more than four weeks after the last dose of medication in a patient who previously had a normal ANC and no other identifiable causes [5]. LON has been identified as a potential adverse effect in patients undergoing treatment with rituximab, a chimeric monoclonal anti-CD20 antibody used in rheumatologic diseases and hematologic malignancies [6]. Before ocrelizumab's availability, rituximab was used off-label for multiple sclerosis, with a few cases reporting LON [7,8]. In a randomized clinical trial, mild neutropenia occurred in 13% of primary progressive multiple sclerosis patients treated with ocrelizumab compared to 10% in the placebo group [9]. However, LON was not reported in relapsing-remitting multiple sclerosis patients using ocrelizumab. More recent articles have documented LON in five cases of ocrelizumab-treated patients, including three with relapsing-remitting and two with primary progressive multiple sclerosis (Table 2) [4,5,10-12].

**Table 2:** Summary of case reports on ocrelizumab-induced neutropenia. MS: Multiple sclerosis; WBC: white blood cell; G-CSF: granulocyte colony-stimulating factor

Author	Patient Age (years), Sex	Dose/Regimen	MS Type	Another Drug Used	Cycles (N)	Time to Neutropenia (days)	WBC	Neutrophil Count	Clinical Status	Action Taken	Recovery Time (days)
Rauniyar et al., 2022 [10].	38, M	600 mg IV infusion every six months.	PP	None	7	90	3.7	0.0	Continuous, low-grade fever, chills, rigor, and painful swelling of the left great toe. Generalized weakness and vesicular lesions in the mouth.	IV antibiotics (vancomycin and piperacillin/tazobactam), acyclovir, and filgrastim (a recombinant GMCSF) 480 mcg.	30
Auer et al., 2020 [4].	21, F	600 mg IV infusion every six months.	RR	None	1	120	2.3	0.3	Asymptomatic.	A prophylactic anti-infective medication with lidaprim and acyclovir.	7
Zanetta et al., 2020 [5].	26, F	600 mg IV infusion every six months.	RR	None	3	75	1.1	0.0	Mouth pain, headache, fever with chills evolving over two days.	Empirically treated with acyclovir 10 mg/kg three times a day and ceftriaxone 2 g/day, IV.	2
Baird-Gunning et al., 2020 [11].	34, M	600 mg IV infusion every six months.	PP	None	1	42	3.4	0.50	Fever with abdominal tenderness.	Broad-spectrum IV antibiotics and G-CSF and ceased oral intake.	5
Cohen et al., 2019 [12].	35, F	600 mg IV infusion every six months.	RR	Armodafinil, vitamin D, norgestimate, estradiol, valacyclovir	2	78	2.5	0.0	Mouth pain, increased fatigue, myalgia, and chills followed by fever evolving over five days.	Empirically treated with prophylactic (cefepime and acyclovir), filgrastim 300 mcg, and methylprednisolone.	3

These cases showed LON onset ranging from 42 to 120 days post-treatment [4,5,10-12], aligning with our report where LON emerged 46 days after the last ocrelizumab dose. We suggest that nitrofurantoin might have exacerbated the neutropenia. It is crucial to exclude other causes of acquired neutropenia, such as vitamin B12 deficiency, viral infections (e.g., Epstein-Barr virus, Parvovirus B19), hematological malignancies (e.g., myelodysplastic syndromes), and immune-mediated destruction [13].

The precise mechanism behind LON remains unclear. However, bone marrow studies suggest an association between LON and a halt in white cell line maturation, potentially due to excess B-cell activating factor, which promotes B-cell recovery. This suggests a diversion of bone marrow activity from granulocyte to B-cell production [6]. In cases of medication-induced neutropenia, a granulocyte colony-stimulating factor like filgrastim may be administered to accelerate recovery by stimulating neutrophil production and activation [14].

## Conclusions

LON is maybe an infrequent but significant side effect of anti-CD20 antibody therapy. This unexpected side effect underscores the necessity of regular blood monitoring for early detection of severe neutropenia. Moreover, it is vital to put in consideration using ocrelizumab in conjunction with drugs known to cause neutropenia, such as nitrofurantoin in this case, when alternative options are available.
